# Spatial Geometry Analysis of Roadside LiDAR for Improved Vehicle Clustering Accuracy

**DOI:** 10.3390/s26134068

**Published:** 2026-06-26

**Authors:** Carolina Fontalvo, Qiyang Luo, Martin Lucero, Keshav Jimee, Rupak Khadka, Mohammad Soltanirad, Tamer Bataineh, Hongchao Liu

**Affiliations:** Department of Civil, Environmental and Construction Engineering, Texas Tech University, Lubbock, TX 79409, USA; carolina.fontalvo@ttu.edu (C.F.); martluce@ttu.edu (M.L.); kjimee@ttu.edu (K.J.); rukhadka@ttu.edu (R.K.); mohammad.soltanirad@ttu.edu (M.S.); tamer.bataineh@ttu.edu (T.B.)

**Keywords:** Roadside LiDAR, unsupervised clustering, beam distribution, tracking performance

## Abstract

Roadside LiDAR is a key sensing technology for intelligent transportation systems (ITSs) due to its high-precision spatial information and reliable monitoring of traffic environments. However, extracting traffic information from LiDAR point cloud data remains challenging because measurements are produced through angular sampling, causing the spacing between adjacent points to depend on radius and beam distribution. This study proposes a geometry-aware framework that incorporates LiDAR sampling geometry into the neighborhood criterion used to determine point-to-point association. The formulation defines neighborhood tolerance as a function of radial distance and vertical angular separation, enabling clustering decisions that are consistent with the sensing mechanism. In addition, the approach integrates deployment constraints based on sensor mounting height and region-of-interest limits to maintain physically meaningful connectivity under roadside sensing conditions. A systematic calibration procedure is conducted to estimate the scaling factor and radial spacing parameters and evaluate the method using both controlled and real-world datasets. Experimental results reveal that the proposed approach improves clustering accuracy and stability by reducing false negatives in sparse regions while avoiding excessive cluster merging in dense areas. The method demonstrates robust performance across varying sensing conditions and achieves higher accuracy than baseline approaches without parameter retuning, while introducing negligible computational overhead.

## 1. Introduction

Infrastructure-based sensing technologies are essential components for intelligent transportation systems (ITSs) as they improve real-time perception in safety monitoring and traffic operations, support cooperative driving environments, and provide valuable information for offline traffic analysis. Compared with other sensing technologies, such as cameras and radar, LiDAR provides high-precision spatiotemporal information from traffic scenes, capturing depth cues of road users, and exhibits robust performance across varying adverse weather and light conditions [1,2,3,4]. The integration of roadside LiDAR in ITSs has gained significant interest due to its superior spatial coverage, enhanced detection and monitoring stability, higher perception accuracy, and reliability of scenes [4,5,6].

Extracting traffic information from roadside LiDAR mainly involves four major tasks: background filtering, object clustering, object classification, and multi-object tracking [4,7]. Moreover, Zhang et al. [6] described additional tasks to be considered, incorporating trajectory reconstruction, speed and motion estimation, and behavior or intention prediction.

Among these stages, object clustering plays a key role in organizing raw point cloud data into meaningful object-level representations. Preserving a larger proportion of points during clustering may be beneficial for subsequent perception tasks as this enhances cluster completeness and improves geometric representation and the quality of data that are used in subsequent stages of the pipeline. Conversely, errors introduced at this stage propagate into classification quality, tracking stability, and trajectory continuity [8]. Therefore, reliable clustering techniques are essential to ensure consistent and accurate perception in roadside LiDAR systems.

Even so, achieving such reliable clustering is inherently influenced by the sensing capabilities of LiDAR devices. The outcome can vary depending on their hardware configuration, particularly the number of laser beams. The number of beams and their distribution determine the spatial resolution, detection range, and point sparsity [4,7,9]. The key challenge is that sparsity is structured by the sensing mechanism. A return at 15 m and a return at 75 m are not sampled under the same geometric sampling density, even when they belong to objects with similar physical size. Recent work on beam design and deployment reinforces that point cloud structure is shaped by both sensor geometry and installation geometry [9,10,11,12].

Since there is currently no standardized LiDAR configuration for roadside sensing applications, many existing perception algorithms are developed and evaluated for a specific sensor type and cannot be directly transferred to systems with different beam distributions or resolutions [4,7]. Existing clustering methods have focused on the Euclidean space and do not explicitly account for angular sampling effects, treating beam angles as independent variables despite their geometric interdependence. This limitation reduces the robustness and transferability of clustering approaches across different sensing setups [4,9].

As a result, the mismatch between sensing geometry and clustering assumptions leads to several practical failure modes, and point density declines with radius; therefore, far vehicles are often represented by sparse or incomplete returns, and occlusion leads to incomplete or missing observations [13,14]. Under such conditions, one vehicle may be over-segmented, creating duplicate detections and unstable tracks [7,9]. Similarly, nearby objects may be under-segmented, compromising object identity and weakening trajectory continuity. Finally, residual points create a third failure mode by inflating false positives. Chen et al. [5] describe the geometric source of this problem: because LiDAR samples through angular beams, the Euclidean spacing between adjacent returns increases with range, so a fixed neighborhood threshold cannot perform consistently across near and far regions. For many devices, the inconsistency is not purely radial. Vertical channel spacing is often nonuniform, so the appropriate clustering tolerance may vary not only with range but also with ring position and beam distribution [10,15].

Beyond these accuracy-related challenges, prior studies have identified clustering as the primary computational bottleneck in LiDAR perception pipelines [5,10,16], since it requires querying neighbor points and performing distance computations across large point cloud datasets. As a result, balancing computational efficiency with detection accuracy remains a critical challenge when processing large-scale LiDAR data. [4,5,13]. Particularly in the clustering stage, maintaining sufficient point density within object clusters and ensuring that meaningful spatial information is preserved are essential for reliable classification, tracking, and trajectory analysis [7,14,17], especially for real-time information delivery and the activation of warning measures within a short response time. Such reliability is particularly critical for high-speed corridors, where extended detection ranges are necessary to provide the lead time required for effective collision avoidance systems.

To address these combined challenges of geometric inconsistency and computational cost, several studies have proposed different methodological approaches. One methodological direction adapts density-based spatial clustering of applications with noise (DBSCAN) methodology by tuning neighborhood parameters across distance or dividing the sensing region into range-dependent areas [18]. Another approach restructures the data into spherical maps, range images, or channel azimuth grids so that neighborhood relations are evaluated in a representation aligned with the acquisition process [5,7,19]. A third methodology introduces resolution-aware weighting within broader perception pipelines, recognizing that LiDAR sampling is not isotropic in Cartesian space [10]. These approaches differ in implementation, but they point to the same practical limitation: clustering quality deteriorates when connectivity is defined without accounting for the sensing geometry that produced the point cloud.

Unlike empirical range-adaptive thresholds or representation-specific clustering schemes, this study derives the neighborhood condition directly from LiDAR angular sampling and roadside deployment geometry. The proposed method formulates point connectivity from radial distance, vertical beam separation, sensor height, and ROI constraints. This allows the same geometry-normalized distance criterion to be integrated into standard unsupervised clustering methods without changing their underlying structure. Therefore, this study proposes a nonuniform, angular-resolution-adaptive clustering framework. The main contributions of this work are threefold as follows:

(1) A geometry-aware adaptive threshold formulation is proposed, wherein neighborhood tolerance is derived from LiDAR sampling geometry through radial distance and vertical angular separation, while being constrained based on sensor mounting height and region of interest (ROI) to ensure that threshold scaling remains physically meaningful under roadside sensing configurations.
(2) A practical integration strategy is introduced to incorporate the adaptive threshold into existing clustering methods through normalized distance evaluation.
(3) A systematic experimental calibration and evaluation of parameters is conducted to estimate the scaling factor and radial spacing and evaluate their impact on fragmentation, cluster merging, cluster completeness, and downstream data quality across DBSCAN and hierarchical clustering techniques.

## 2. Related Work

Roadside LiDAR perception is now supported by a reasonably consistent processing architecture, yet clustering remains one of its least stable stages. This stage carries significant weight because segmentation determines the object boundaries on which subsequent processing depends. A tracker may not restore a clean identity when one vehicle has already been split into several fragments, nor can it easily recover when adjacent actors have been merged before association begins.

The roadside setting magnifies this difficulty. Far-range returns, frequent occlusion, and incomplete object surfaces are common in infrastructure sensing. They can cause discontinuous tracks and unstable speed estimates, which further motivates preserving complete and physically meaningful clusters before the tracking stage. Chen et al. [20] documented these effects in a roadside LiDAR framework for pedestrian and vehicle detection and tracking, showing that incomplete clusters and mutual occlusion complicate both segmentation and trajectory extraction. Chen et al. extended that argument by demonstrating why fixed Euclidean thresholds fail under roadside sampling geometry [5]. The distance between adjacent returns grows with range, so a threshold that preserves point association in the near field can miss portions of the same object at longer distances, while a threshold large enough for the far field can merge distinct nearby targets. That geometric instability sits at the center of the clustering problem.

A large share of the literature has been addressed through density-based clustering, especially DBSCAN and its variants. DBSCAN remains attractive because it can recover arbitrarily shaped clusters, and it labels isolated points as noise [21]. These strengths explain the continued implementation in roadside LiDAR pipelines [22,23]. However, its limitations are equally well known. Performance depends on the neighborhood radius and minimum point count, and those parameters are difficult to hold fixed when point density changes substantially across the scene [24]. Zhang et al. [25] partly addressed this by incorporating area-of-interest selection, ground-point removal, vehicle clustering, and tracking, demonstrating that clustering-based processing can support object detection under complex traffic conditions. Wang et al. [18] proposed automatic epsilon estimation to reduce manual tuning. Such approaches improve usability and adaptability, but they still adapt to observed density rather than normalizing the geometric source of density variation.

Another body of work uses the acquisition structure of LiDAR more directly. These methods project the point cloud data into a spherical map, range image, or related structured representation and then evaluate point association through local searches in that structured domain rather than repeated Euclidean neighborhood queries in unstructured three-dimensional space. The gain is not only computational. A structured representation preserves sensor topology and makes adjacency relations more faithful to the scanning process. Bogoslavskyi and Stachniss demonstrated this for sparse three-dimensional laser scans [19]. Chen et al. carried the same logic into roadside LiDAR through FSPC, where clustering is performed on a spherical projection with a two-dimensional search window [5]. These studies matter because they move clustering closer to sensing geometry instead of treating the point cloud as an ordinary Cartesian sample.

Roadside-specific work has taken a similar direction. Zhang et al. [7] developed Counted Region Growing by converting roadside LiDAR data into a channel azimuth representation and combining region growing, connected component labeling, and a merge stage designed for computational efficiency. That contribution is valuable for two reasons. It is explicitly tailored to infrastructure sensing, and it indicates that clustering speed can improve substantially when the representation matches the scanning mechanism of the device. Nevertheless, connectivity limitation remains, as it still depends on thresholds selected empirically for the application context and fixed neighborhood rules. The data structure is improved, but the thresholding logic remains only partly tied to the measurement process itself.

The same limitation appears in more conventional Euclidean and region-growing approaches. D’Arco et al. [14] review partition-based, density-based, and hierarchical clustering as common options for roadside object detection, noting that DBSCAN is effective only where local density gradients are manageable and that hierarchical clustering can become expensive on dense point clouds. This is consistent with the broader clustering literature, which highlights that hierarchical methods strongly depend on the choice of distance or dissimilarity metric and require efficient implementations as dataset size increases [26].

More general point cloud segmentation studies reach a related conclusion. Fixed-distance Euclidean clustering is simple and interpretable, but it becomes unstable under strong density variation. Region growing methods depend heavily on seed selection and growth criteria that often require re-tuning across datasets. In each case, the method is forced to interpret nonuniform angular sampling with an association rule that is effectively uniform in Cartesian space.

Some studies have tried to repair that weakness by adapting the threshold itself. Wen et al. proposed an improved Euclidean clustering method in which the distance threshold varies with target distance through a fitted relation [27]. Lin et al. developed an adaptive framework that assigns each point a local threshold derived from geometric characteristics rather than a scene-wide constant [3]. These studies move beyond a single global radius and recognize that tolerance must vary across the point cloud. Their limitation is conceptual rather than practical. The threshold is still driven by empirical fitting, local statistics, or scene-level geometric heuristics rather than by a deterministic expression derived from LiDAR sampling intervals. That distinction is important because a geometry-derived tolerance can be interpreted as a property of the sensing process rather than as a parameterized response to a particular scene.

A closely related idea appears in learned perception pipelines. RangeSeg and improved Euclidean clustering methods are especially relevant because they do not treat instance grouping as a generic postprocessing step. After semantic segmentation on the range image, these methods cluster points with a resolution-aware DBSCAN distance that reflects uneven LiDAR sampling [10,27,28]. This suggests a useful precedent: clustering improves when the metric accounts for sensor resolution structure rather than relying on an isotropic Cartesian norm.

However, these approaches differ from the present study in two important ways. First, RangeSeg embeds the grouping procedure within a supervised segmentation architecture, whereas the proposed framework is designed for unsupervised roadside clustering. Second, adaptive Euclidean or resolution-aware methods typically modify the clustering distance using empirical range relations, learned features, or local resolution adjustments, while the present method explicitly derives the neighborhood tolerance from the LiDAR angular sampling geometry and roadside deployment constraints. Therefore, the contribution is not only the use of an adaptive distance but the formulation of a general geometry-normalized association rule that can be applied to standard clustering methods.

Recent beam design and deployment studies have sharpened motivation further. He et al. showed that vehicle perception quality changes materially with beam distribution, installation height, and tilt angle [9,11,29]. Similarly, Ge et al. [30] developed a three-dimensional blind-zone simulation model for roadside LiDAR deployment, showing that detection performance depends on sensor configuration, static infrastructure conditions, and dynamic occlusion during live traffic. Previous works [12] likewise found that perception performance is linked to point cloud density and uniformity and that inappropriate tilt can degrade sensing quality through uneven spatial distribution. These studies are not clustering-focused, but they establish an important condition for clustering research: the point cloud structure is partly determined upstream by the sensor’s internal beam distribution and by roadside deployment geometry. These findings indicate that clustering rules that ignore beam distribution may have limited generalizability across roadside LiDAR configurations.

The remaining gap is specific. Existing roadside approaches have improved clustering through range-adaptive DBSCAN variants, structured representations, empirically adaptive thresholds, and resolution weighting inside learned systems [5,7,10,27]. However, a general association rule derived directly from LiDAR sampling geometry and applicable across standard unsupervised clustering methods remains underdeveloped. This is the position of the present study. It frames clustering as a geometry-normalized point-association problem and incorporates sensing radius, mounting height, and ROI limits into the neighborhood threshold itself. In this way, beam configuration and deployment geometry are not treated only as external factors affecting perception quality, but as variables that define the clustering criterion.

Under this perspective, the central question is no longer how to re-tune a global radius for each scene but how to define connectivity so that it remains consistent with the sensing process that generated the observations.

## 3. Methodology

**Figure 1:**
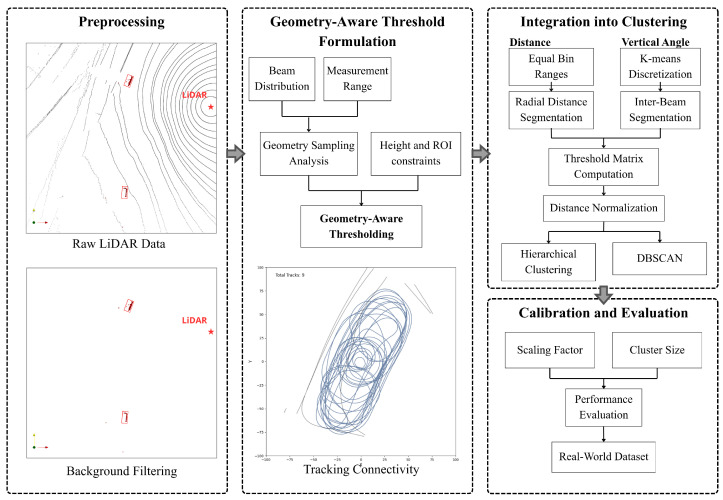
Proposed geometry-aware clustering framework. The star symbol represents the LiDAR position, and the bounding boxes indicate the detected vehicles.

The proposed framework comprises four main stages: data preprocessing, geometry-aware threshold formulation, integration into clustering, experimental calibration and evaluation, as illustrated in Figure 1. In the preprocessing stage, raw LiDAR point cloud data are filtered to remove background structures and isolate foreground objects within the ROI. The formulation stage derives adaptive neighborhood tolerances based on LiDAR sampling properties, including beam distribution, sensing range, and sensor deployment constraints. The framework takes this idea into practice by converting geometry-derived spacing into a threshold matrix that can be used for normalized pairwise distance evaluation.

Specifically, the formulation accounts for the nonuniform spatial structure of LiDAR measurements by linking the neighborhood criterion to vertical angular separation and radial distance. In the following stage, adaptive thresholds are integrated into standard clustering methods through distance normalization, enabling consistent connectivity across varying point densities without modifying the underlying clustering algorithms. The framework is further calibrated by selecting the scaling factor λ and radial spacing Δr and it is evaluated using real-world datasets to assess clustering performance and connectivity preservation.

### 3.1. Initial Preprocessing

**Figure 2:**
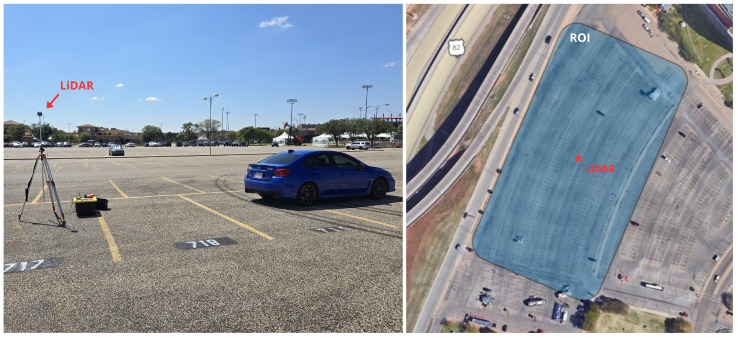
Study area and LiDAR system setup. The star symbol represents the LiDAR position, and the blue area indicates the region of interest.

A controlled dataset was collected using a Velodyne VLP-32C LiDAR sensor, whose specifications are detailed in the user manual [31], deployed in an open parking environment, as illustrated in Figure 2. The setup was designed to minimize occlusion while capturing near and far-field observations. To minimize confounding factors such as multi-target interference, a 15 min dataset was recorded involving a reference vehicle executing diverse driving patterns. This recording encompasses variable speeds, straight motion, turning motion, and short stationary periods, ensuring that the algorithm is exposed to a wide range of sensing radii and viewing angles.

Ground truth for the controlled dataset was established through manual frame-level annotation of the LiDAR point cloud sequence. Although the controlled scenario primarily involved a single reference vehicle, each frame was reviewed to verify the number of valid objects within the ROI and confirm the identity of the reference vehicle. These annotated frame-level object counts were used as the reference values for the MARE calculation in Section 4.1.

The background removal technique implemented follows the approach proposed by Zhang et al. [32]. The process begins with background dataset construction, where multiple frames are aggregated to build a background dataset by identifying the farthest or mean distance recorded for each specific angular pair. The background filtering process is subsequently performed by comparing the data of the current frame with the background data set; points are extracted as potential vehicles or pedestrians if the distance difference at the same angular coordinate exceeds a predefined threshold.

### 3.2. LiDAR Sampling Geometry and Threshold Formulation

**Figure 3:**
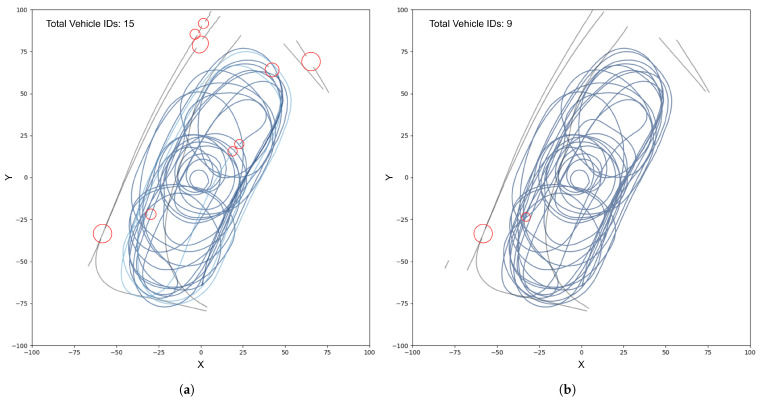
Tracking Continuity from Hierarchical clustering. (**a**) Fixed threshold on the controlled dataset. (**b**) Geometry-Adaptive Threshold on controlled dataset. The blue line indicates the track of the reference vehicle, and the red circles represent the track discontinuities.

A fundamental limitation in LiDAR clustering is the loss of grouping consistency across sensing distances. As radial distance increases, point density decreases, and inter-point spacing grows, often exceeding the fixed Euclidean thresholds used in conventional clustering methods. Consequently, points belonging to the same object may fail to satisfy the neighborhood condition, leading to artificial fragmentation into multiple clusters. This effect is illustrated in Figure 3a through a representative case in which the trajectories of vehicles are segmented into multiple disconnected clusters under a standard clustering method, highlighting the limitations of fixed-distance association in nonuniform LiDAR data.

For roadside LiDAR clustering, admissible connectivity should be scaled with the same geometry that determines local inter-point spacing. The VLP-32C sensor (Velodyne Lidar, San Jose, CA, USA) used in this study samples the scene through fixed azimuth rotation and discrete vertical channels. This behavior arises from angular sampling, where adjacent returns are separated by angular increments, resulting in larger Euclidean distances between neighboring points at greater sensing ranges. Moreover, due to the nonuniform vertical beam distribution of the sensor, the vertical angular separation varies across channels. In particular, beams at extreme vertical angles exhibit larger inter-beam separations, which further amplify inter-point spacing compared to central channels.

LiDAR angular sampling and beam distribution define the spatial structure of the point cloud, thereby influencing perception quality. He et al. [9] reported that uniform angular beam spacing tends to favor near-field vehicle perception, whereas uniform sensing distance distributions, also known as nonuniform beam distribution, improve detection performance for distant targets. Similarly, studies on LiDAR deployment have confirmed that perception performance depends on the number and distribution of beams intersecting the ROI, highlighting that traffic visibility is determined not only by object position but also by sensing geometry. Accordingly, this study formulates the inter-point spacing as a function of sensing range and vertical beam separation.

**Figure 4:**
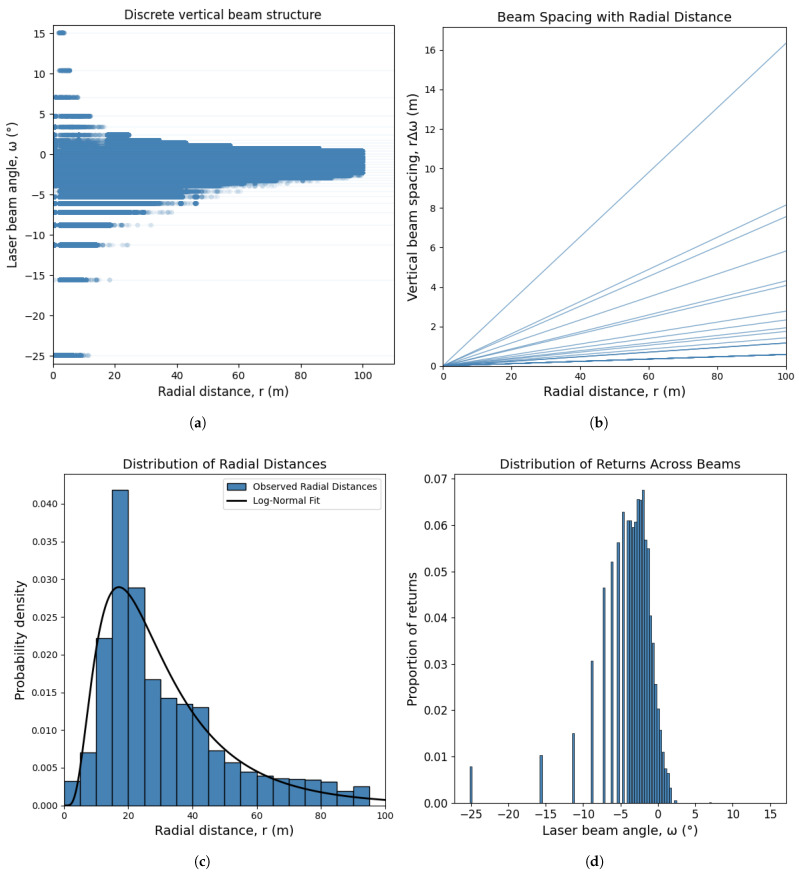
Sampling structure and beam–spacing imbalance of the foreground point clouds. (**a**) Discrete Vertical Beam Structure. (**b**) Beam Spacing with Radial Distance. (**c**) Distribution of Radial Distances (**d**) Distribution of Returns Across Beams.

The empirical sampling geometry of the point cloud data acquired by the Velodyne VLP-32C LiDAR, after processing the background filtering step, is illustrated in Figure 4. Figure 4a shows that the returns are organized along discrete vertical beam angles, reflecting the structured sampling pattern of the sensor. Figure 4b shows the projected vertical beam spacing, which increases with radial distance and varies across beam pairs due to the nonuniform vertical beam distribution. This behavior implies that the projected inter-point spacing also varies across the scene.

The distributions in Figure 4c,d characterize the imbalance of the filtered point cloud. Radial distance is heavily concentrated in the near-field, while the vertical angular distribution is highly skewed between −5° and 0°. This concentration of returns indicates that most potential clusters originate from a narrow range of sensing geometries. Consequently, the filtered point cloud exhibits a highly nonuniform spatial density, motivating the use of range-dependent thresholding and beam-spacing-based angular grouping.

As a result, relying on a global Euclidean constant becomes insufficient. These observations motivate the use of a clustering threshold that adapts to the local sensing geometry. Based on this, the following sections detail the proposed modeling framework, with the notation summarized in Table 1.

For neighboring LiDAR returns, we define the sampling geometry by a set of vertical and horizontal beam angles, denoted in Equations (1) and (2), respectively. The corresponding angular spacings between consecutive beams are defined in Equations (3) and (4).

(1) $$\begin{array}{c} \omega =\left\{\omega_{j}|\omega_{j}\in \left[\omega_{\min},\omega_{\max}\right]\right\},\;j=1,2,\ldots ,N_{\omega } \end{array}$$

(2) $$\begin{array}{c} \alpha =\left\{\alpha_{i}|\alpha_{i}\in \left[0,2\pi \right),\;i=1,2,\ldots ,N_{\alpha }\right\} \end{array}$$

(3) $$\begin{array}{c} \Delta \omega_{j}=\left|\omega_{j+1}-\omega_{j}\right|,\;j=1,2,\ldots ,N_{\omega }-1 \end{array}$$

(4) $$\begin{array}{c} \Delta \alpha_{i}=\left|\alpha_{i+1}-\alpha_{i}\right|,\;i=1,2,\ldots ,N_{\alpha }-1 \end{array}$$

Similarly, Equation (5) expresses the local spacing, decomposed into horizontal, vertical, and radial components, based on the spherical conversion of Euclidean distance. However, in mechanical rotating LiDAR configurations, azimuth resolution Δα is fixed (where $\Delta \alpha_{i}=\Delta \alpha$) and typically ranges from $0.1^{{}^{\circ}}$ to $0.4^{{}^{\circ}}$ [4,33]. In the present study, the azimuth resolution is fixed at $0.2^{{}^{\circ}}$ ($3.49\times 10^{-3}$ rad), which is small relative to the vertical beam separations Δω considered in the threshold formulation. Because the horizontal term enters as the squared term ${\left(r_{i}\Delta \alpha \right)}^{2}$, its relative effect is further reduced compared with larger vertical spacing terms. Therefore, the dominant geometry-dependent variation is approximated by the product of radial distance and vertical angular separation, as expressed in (6).

(5) $$\begin{array}{cc} d_{i} & \approx \sqrt{{\left(r_{i}\Delta \alpha \right)}^{2}+{\left(r_{i}\Delta \omega_{j}\right)}^{2}+\Delta r^{2}} \end{array}$$

(6) $$\begin{array}{cc} d_{i}^{2} & \propto {\left[r_{i}\Delta \omega_{j}\right]}^{2}+\Delta r_{i}^{2} \end{array}$$

Since the sensor is mounted above ground and clustering is restricted to a predefined ROI, the threshold must also respect the deployment geometry. The maximum valid radius associated with each beam is defined by the intersection between the direction of the beam and the vertical ROI limit, as given in Equation (8). For each pair of adjacent beams, the admissible range $r_{j,m}^{eff}$ is conservatively limited by the minimum of the two valid radii (9). This ensures that the threshold scaling remains physically meaningful within the sensing region. This constraint could also prevent under-segmentation caused by larger thresholds. The geometry-aware thresholding equation is finally defined in (10).

(7) $$\begin{array}{cc} r_{m} & \in \left\{d_{1},d_{2},\ldots ,d_{M}\right\},\;m=1,2,\ldots ,M \end{array}$$

(8) $$\begin{array}{cc} r_{j}^{\max} & =\left\{\begin{array}{cc} \frac{z_{\mathrm{ROI}}^{\max}-h_{L}}{\tan(\omega_{j})}, & \omega_{j}>0,\\ \frac{z_{\mathrm{ROI}}^{\min}-h_{L}}{\tan(\omega_{j})}, & \omega_{j}<0,\\ r_{\mathrm{ROI}}, & \omega_{j}=0, \end{array}\right. \end{array}$$

(9) $$\begin{array}{cc} r_{j,m}^{\mathrm{eff}} & =\min\;\left(r_{m},\;r_{j}^{\max}\right) \end{array}$$

By this formulation, the clustering threshold is no longer imposed through a single global Euclidean radius. Instead, it is tied directly to the LiDAR sampling geometry and the deployment constraints that generated the observations. The resulting threshold values are then grouped and integrated into existing clustering methods through normalized distance evaluation, as described in the following section.

(10) $$t_{j,m}=\lambda \;\left[\Delta \omega_{j}^{2}\;r_{j,m}^{\mathrm{eff}(2)}+\Delta r^{2}\right]$$

### 3.3. Threshold Integration Strategy

To construct adaptive threshold groups, the radial domain is divided into equal-width intervals (Equation (7)) because the projected inter-point spacing increases approximately linearly with sensing distance within a fixed angular group. For vertical angular separations, however, the empirical distribution is discrete and nonuniform, as shown in Figure 4d.

This nonuniformity arises from two sources: the sensor beam layout produces uneven vertical angular intervals, and the filtered point cloud contains an uneven number of returns associated with each angular separation. Therefore, standard quantile-based discretization may produce groups with balanced sample counts but physically inconsistent angular ranges. To address this issue, vertical angular separations were partitioned using one-dimensional k-means clustering, a well-established method for grouping values by minimizing within-group variance and providing efficient discretization of nonuniform distributions [34].

(11) $$\begin{array}{cc} T_{g,m} & =\max_{\Delta \omega_{j}\in G_{g}}\left(t_{j,m}\right) \end{array}$$

(12) $$\begin{array}{cc} G_{\omega } & =\left\{G_{1},G_{2},\ldots ,G_{K}\right\},\;\Delta \omega_{j}\in G_{g} \end{array}$$

The resulting thresholds are organized into a matrix *T*, where each element $T_{g,m}$ represents the maximum admissible squared Euclidean separation for a given angular group *g* and radial interval *m*, as computed in Equation (11). To ensure compatibility with standard clustering algorithms that typically rely on a single global parameter, we employ distance normalization. In this formulation, the squared Euclidean distance $d(p_{i},p_{k})$ between two points is scaled by the corresponding threshold from *T*:

(13) $$\begin{array}{cc} d_{\mathrm{norm}}\left(p_{i},p_{k}\right) & =\frac{d(p_{i},p_{k})}{T_{g,m}} \end{array}$$

(14) $$\begin{array}{cc} d_{\mathrm{norm}}\left(p_{i},p_{k}\right) & \leq 1 \end{array}$$

This normalization allows the adaptive thresholds to regulate connectivity while preserving the core computational structure of algorithms such as DBSCAN or Hierarchical Clustering. The complete procedure for generating the threshold matrix is detailed in Algorithm 1.
**Algorithm 1** Clustering with Geometry-Aware Normalization.**Input:** ω, ${\left\{r_{m}\right\}}_{m=1}^{M}$, $h_{L}$, $z_{\mathrm{ROI}}$, λ, Δr, *K*, Foreground point cloud $𝒫={\left\{p_{i}\right\}}_{i=1}^{N}$, **Output:** cluster labels per frame
1:Compute angular separations: $\;\Delta \omega_{j}\leftarrow \left|\omega_{j+1}-\omega_{j}\right|,\;j=1,\ldots ,N_{\omega }-1$2:Partition $\Delta \omega_{j}$ into *K* groups using 1D k-means: $\;\Delta \omega_{j}\in G_{g}$3:Compute maximum valid radius for each beam: $\;r_{j}^{\max},\;j=1,\ldots ,N_{\omega }$4:Compute admissible pair radii for adjacent beams (j,j+1):     $\;{\bar{r}}_{j}^{\max}\leftarrow \min\left(r_{j}^{\max},r_{j+1}^{\max}\right),\;j=1,\ldots ,N_{\omega }-1$5:**for** each beam pair *j* **do**6:    **for** each radial bin $r_{m}$ **do**7:        $r_{j,m}^{\mathrm{eff}}\leftarrow \min\left(r_{m},{\bar{r}}_{j}^{\max}\right)$8:        $t_{j,m}\leftarrow \lambda \cdot [\Delta \omega_{j}^{2}\;r_{j,m}^{\mathrm{eff}(2)}+\Delta r^{2}$]9:    **end for**10:**end for**11:Aggregate thresholds within each group: $\;T_{g,m}\leftarrow \max_{\Delta \omega_{j}\in G_{g}}\left(t_{j,m}\right)$12:**for** each point cloud $p_{i}$ **do**13:    Sort points by azimuth and laser ID14:    Assign each point an angular group $g_{i}$ from its vertical angle15:    Assign each point a radial bin $m_{i}$ from its range16:    Retrieve pointwise multiplier: $\tau_{i}\leftarrow T_{g_{i},m_{i}}$17:    Compute pairwise squared Euclidean distances: $d^{2}\left(p_{i},p_{k}\right)$18:    **for** each pair (i,k) **do**19:        $\tau_{ik}\leftarrow \min\left(\tau_{i},\tau_{k}\right)$20:        Normalize pairwise distance: $d_{\mathrm{norm}}\left(p_{i},p_{k}\right)\leftarrow \frac{d^{2}\left(p_{i},p_{k}\right)}{\tau_{ik}}$21:    **end for**22:    Apply clustering using the normalized pairwise distances23:    Compute cluster sizes and discard clusters with fewer than $P_{\min}$ points24:**end for**25:**return** valid cluster labels


### 3.4. Calibration Parameter Selection and Experimental Evaluation

Two key parameters govern the behavior of the adaptive formulation. The first is the scale factor λ, which controls the magnitude of the threshold derived from the geometry law (Equation (11)). The second is the radial spacing Δr, representing the allowable radial variation between neighboring returns from the same object. In this study, Δr is treated as a tunable calibration parameter that accounts for object-depth variations not fully captured by the angular-spacing term. Together, they balance cluster completeness against false merging and noisy residual clusters.

These parameters were calibrated using a 15 min controlled dataset, in which a single vehicle executed near-field and far-field passes, straight motion, turning motion, and short stationary periods. That design isolated clustering behavior from heavy multi-object interference while still exposing the method to a wide range of sensing radius and viewing angles.

The performance of the proposed adaptive criterion is benchmarked using unsupervised clustering methods, DBSCAN and Hierarchical Clustering. To assess the generalization of the calibrated parameters, the model is further validated using an independent dataset collected from regular signalized intersections. This validation phase utilized complex traffic scenarios to confirm the effectiveness of the proposed method in high-density, multi-object environments.

Fragmentation is defined as the partition of one physical vehicle into multiple disjoint clusters within a frame. Merging is defined as the unintended union of distinct physical objects into one cluster. Completeness is evaluated as the ability to preserve valid object returns that would otherwise be discarded under a fixed threshold rule. Because fragmentation and merging can offset each other in simple count totals, the reported performance package used two complementary indicators: cluster accuracy and the percentage of points gained overall. The latter metric specifically validates cluster completeness by measuring the retrieval of valid object returns that would otherwise be lost under a fixed-distance threshold.

**Figure 5:**
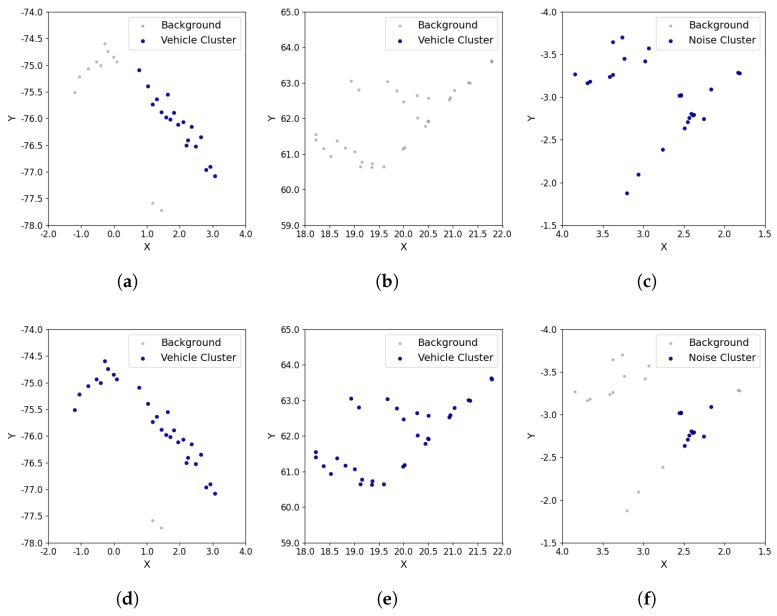
Effects of geometry–aware normalization in clustering scenarios. (**a**) Baseline: Missed Detection. (**b**) Baseline: Fragmented Cluster. (**c**) Baseline: Noise Cluster. (**d**) Proposed: Recovered Cluster. (**e**) Proposed: Merged Cluster. (**f**) Proposed: Noise Suppression.

Therefore, the evaluation focuses on the joint behavior of cluster integrity and practical runtime. By systematically analyzing these metrics across both controlled and intersection datasets, this evaluation examines how the proposed formulation preserves cluster integrity across varying sensing ranges without incurring excessive computational overhead. Three representative clustering scenarios observed during calibration are presented in Figure 5 to illustrate the effect of the proposed method. In the first case, conventional clustering fails to detect a vehicle due to insufficient point association, whereas the proposed method successfully recovers most of the points in the object. In the second case, fragmented clusters produced at larger sensing distances are merged into a single vehicle cluster. In the third case, spurious clusters caused by noise in near-field regions are reduced or suppressed, improving the robustness to false positives. These cases highlight the ability of geometry-aware normalization to adapt connectivity across varying spatial sampling conditions.

## 4. Results

### 4.1. Calibration on Experimental Scenario

The calibration of the geometry-aware model was carried out with a controlled data set from a VLP-32C LiDAR, designed to isolate the clustering behavior from sensor-specific artifacts. These data provide a range of spatial configurations and point density conditions while minimizing environmental variables such as occlusion. By leveraging this approach, this study establishes a baseline to evaluate the sensitivity of the hyperparameter involved in the proposed formulation.

A hyperparameter calibration was conducted on the scaling factor λ and the radial spacing Δr to determine the optimal configuration for the specific LiDAR beam distribution used in this study. The selection process aims to balance object separation in high-density regions, cluster integrity preservation in sparse areas, and ensure that low-density environmental noise is not clustered.

To evaluate performance, the Mean Absolute Relative Error (MARE) of the cluster count was analyzed in a range of values for the scaling factor λ and the radial spacing Δr. This metric quantifies the average magnitude of the deviations between the estimated cluster counts and the ground truth across all frames.

**Figure 6:**
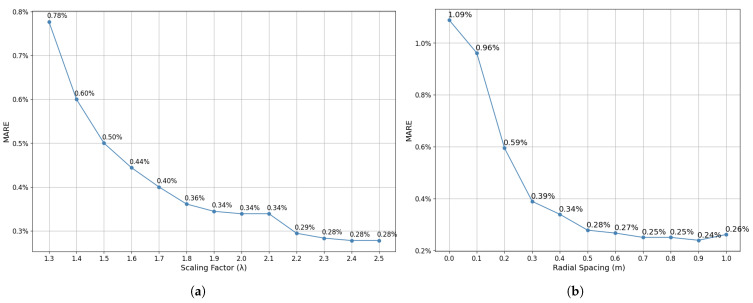
Cluster Mean Absolute Error. (**a**) Sensitivity of Cluster Count Error to λ. (**b**) Sensitivity of Cluster Count Error to Δr.

As illustrated in Figure 6a, the sensitivity of the clustering error to λ exhibits a characteristic curve where the error decreases as the scaling factor facilitates better object grouping in sparse regions, eventually reaching a stabilized plateau. Similarly, Figure 6b displays the response of the error to variations in Δr, highlighting its secondary but essential role in maintaining structural stability in regions with significant radial variation. Together, these plots characterize the trade-off between over-segmentation and under-segmentation, providing a clear mathematical basis for identifying the configurations that minimize counting inconsistencies throughout the experimental sequence.

The results demonstrate that smaller values of λ lead to increased error due to over-segmentation, particularly in the far-field, due to insufficient connectivity between points. In contrast, higher values of λ reduce fragmentation but increase the risk of missing clusters in dense regions. The optimal configuration was observed within the range λ∈[1.7,2.4], resulting in a MARE below 0.40%.

The contribution of Δr was also evaluated. The results indicate that while it plays a secondary role compared to the angular spacing, it provides additional stability in regions with significant radial variation. Although lower error values were observed for larger Δr, the value Δr=0.4 was selected as a more conservative choice, as higher values can increase the risk of cluster merging in operational scenarios with high traffic density. These observations are also reflected in the results summarized in Table 2.

In addition, the computation time reported in Table 2 corresponds to the threshold lookup, normalized distance computation, clustering, and post-processing of small clusters. All methods were benchmarked on the same desktop workstation equipped with an Intel(R) Core(TM) i9-14900K CPU at 3.20 GHz with 64.0 GB RAM, running a 64-bit Windows operating system.

Although under-segmentation is a theoretical risk of adaptive thresholds, it was not observed in this study due to the controlled nature of the dataset and the low traffic volume, which prioritized the evaluation of point association in sparse conditions. In contrast, the main error mode observed in the fixed-threshold approach was the failure to form valid clusters in the far field, leading to an increase in missed detections. As inter-point spacing increases with distance, the fixed neighborhood radius becomes insufficient relative to the sparse spatial sampling, causing the algorithm to perceive distant vehicle returns as isolated points rather than coherent objects.

### 4.2. Clustering Performance Evaluation

The clustering outputs were evaluated across the temporal sequence to assess stability over time. The fixed-threshold approach exhibits more frequent deviations from the true object count between consecutive frames, particularly in later frames where the vehicle remains in regions with lower sampling density. In contrast, the geometry-aware method produces more stable cluster counts that closely follow the ground truth throughout the sequence. This behavior reflects the ability of the adaptive formulation to maintain consistent connectivity across varying spatial conditions. Its stability is essential for applications such as tracking, where reliable temporal continuity is required.

**Figure 7:**
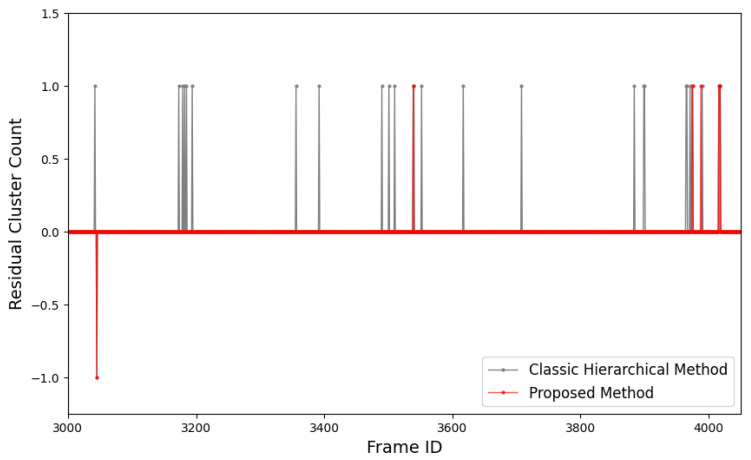
Residual Cluster Count Error for Hierarchical Clustering.

Figure 7 presents a comparison between clustering outputs obtained using hierarchical clustering with a fixed Euclidean threshold and the proposed geometry-adaptive threshold. The fixed-threshold method achieves a baseline accuracy of 95.64%, while the adaptive method improves this performance to 99.51%. The improvement is primarily driven by a reduction in clustering errors in sparse regions, where the adaptive threshold expands the effective neighborhood radius to compensate for reduced point density.

### 4.3. Evaluation on Intersection Datasets

**Figure 8:**
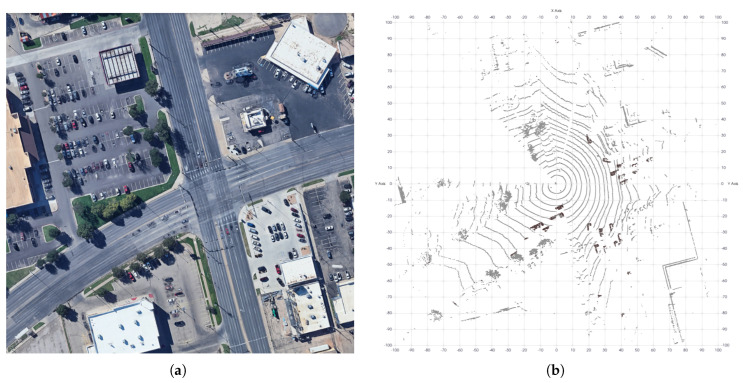

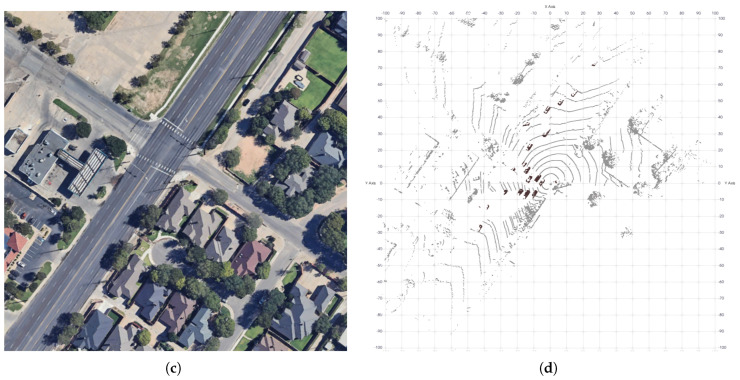
Intersection datasets used in this study. (**a**) Satellite view of intersection A: 50th St. & Ave. Q, Lubbock, TX, (**b**) LiDAR view of intersection A, (**c**) Satellite view of intersection B: 4th St. & Toledo Ave., Lubbock, TX, (**d**) LiDAR view of intersection B.

To validate robustness beyond controlled maneuvers, the framework was evaluated in two complex intersection environments (Figure 8). These datasets introduce additional challenges, including varying traffic density, occlusions, and environmental noise.

The resulting clusters exhibited improved spatial coherence, which translated into enhanced tracking continuity in dynamic traffic scenarios. Importantly, the method demonstrated strong generalization capability without requiring hyperparameter retuning across different intersection environments.

**Figure 9:**
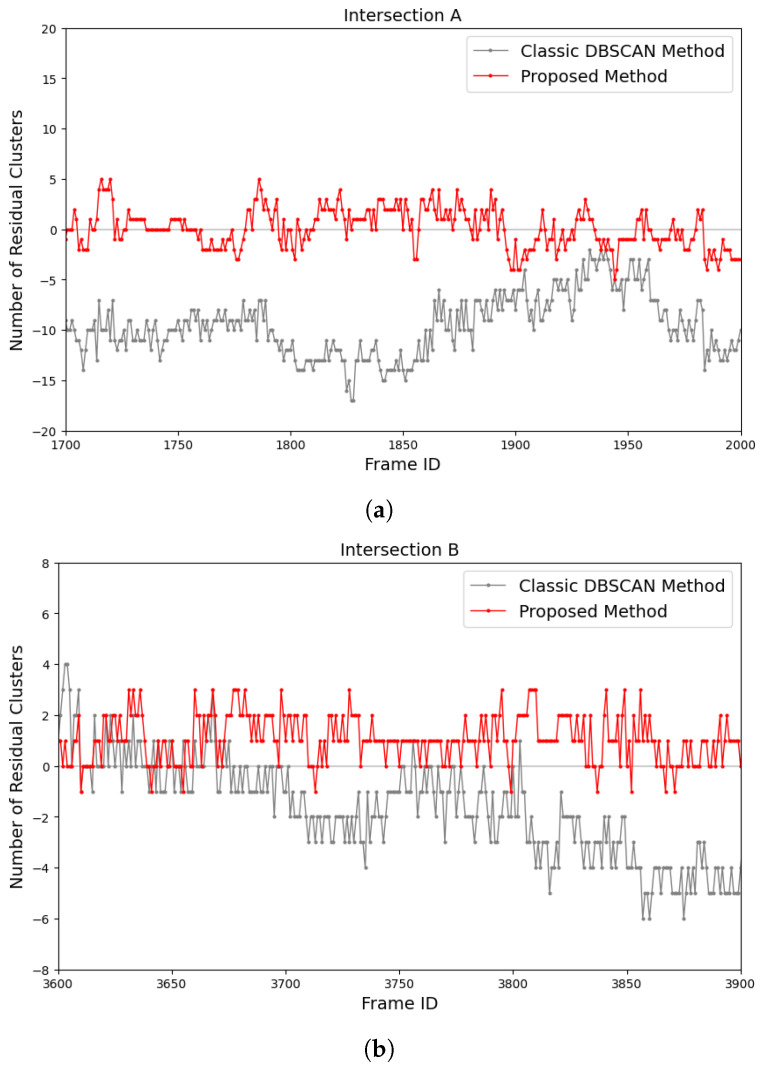
Cluster count residual comparison between classic DBSCAN and the proposed method on intersection datasets. (**a**) Intersection A. (**b**) Intersection B.

In the intersection datasets, Figure 9 illustrates the stability of the proposed method implemented with DBSCAN clustering, as evidenced by the reduced residuals compared to the classic approach at both intersections. The method achieved accuracies of 90.82% and 84.00% for the two locations, respectively, compared to 86.61% and 83.86% obtained by the baseline hierarchical clustering approach. These results confirm that the proposed formulation provides a robust and scalable solution for clustering in heterogeneous LiDAR environments, maintaining high performance across both experimental and operational conditions.

### 4.4. Effect on Tracking Continuity Performance

The impact of the clustering approach on tracking performance was evaluated by analyzing track continuity and fragmentation metrics, MOTA (Multiple Object Tracking Accuracy). Improved clustering consistency directly translates into more stable object trajectories, as the likelihood of cluster splitting or merging across frames is reduced.

**Figure 10:**
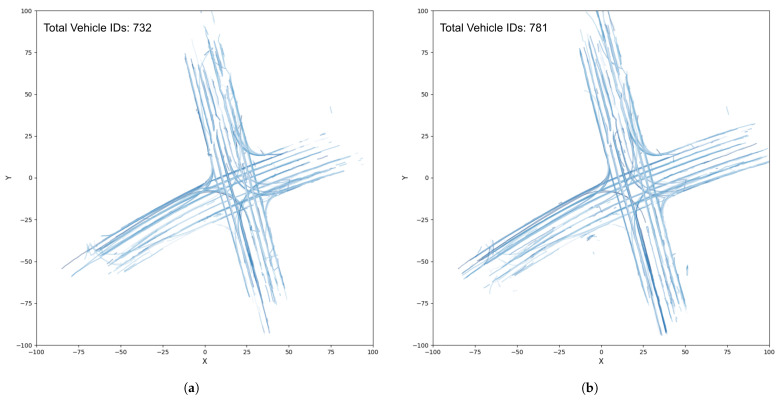
Tracking Continuity from Hierarchical clustering. (**a**) Fixed Threshold on intersection A. (**b**) Geometry-Adaptive Threshold on intersection A.

The impact of enhanced clustering on downstream LiDAR perception is visualized in the tracking trajectories of Figure 3 and Figure 10. The standard method (Figure 3a) results in fragmented, inconsistent paths due to unstable cluster centroids. The geometry-aware formulation (Figure 3b) produces continuous, smooth trajectories, demonstrating superior spatial consistency across the entire 100 m range. The dataset and Python script used to reproduce the tracking performance visualization of controlled and intersection A scenarios are provided in the Supplementary Materials.

As shown in Figure 10a, conventional clustering produces fragmented and discontinuous trajectories, particularly in regions with sparse sampling and varying point density. In contrast, Figure 10b demonstrates that the geometry-aware formulation yields more continuous and coherent tracks by adapting the neighborhood criterion to the LiDAR sampling structure. Although the proposed method produces a higher total number of tracks, this increase is primarily attributed to temporary trajectory discontinuities caused by occlusions and partial observations, rather than artificial cluster splitting or noise amplification. This behavior indicates that the proposed approach preserves the structural integrity of detected objects while improving connectivity across challenging sensing conditions.

Table 3 summarizes the tracking-level performance obtained from the clustering outputs. The geometry-aware formulation improves MOTA for both hierarchical clustering and DBSCAN, mainly by reducing false negatives and false positives. For hierarchical clustering, MOTA increases from 0.9849 to 0.9969, while false negatives decrease from 113 to 27, and identity switches are eliminated. For DBSCAN, MOTA increases from 0.7664 to 0.9598, with a substantial reduction in both false negatives and false positives.

**Figure 11:**
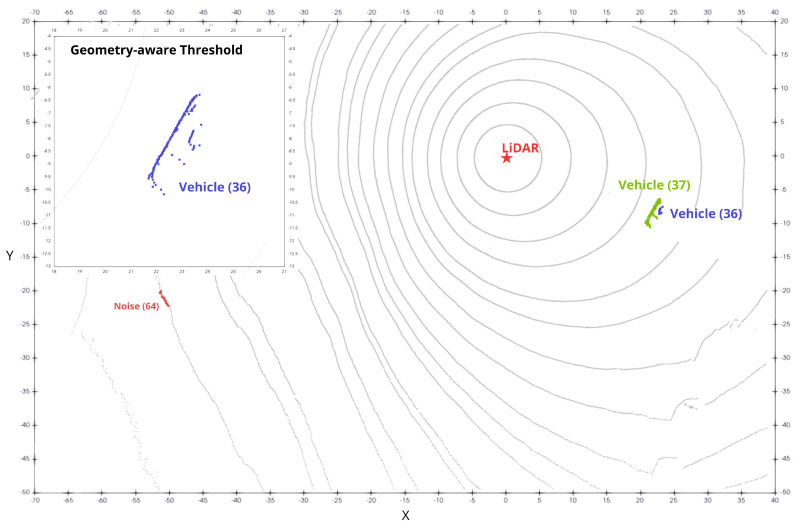
Over–Segmentation Comparison on Frame 8942: Classical vs. Geometry-Aware Hierarchical Methods. The star symbol represents the LiDAR position.

Figure 11 displays a comparative frame analysis using Hierarchical Clustering. In the standard approach, the target vehicle is fragmented into two distinct clusters (IDs 36 and 37), and distant noise (ID 64) is erroneously preserved. Conversely, the proposed formulation unifies the vehicle into a single cluster (ID 36) and increases the spatial density retention by 11.9% (from 109 to 122). Therefore, cluster integrity is maintained in sparse regions without triggering the widespread merging of adjacent objects in the near field.

## 5. Discussion

Incorporating LiDAR sampling geometry into the clustering process significantly improves clustering performance, particularly in maintaining consistency across varying ranges and viewing angles. The results indicate that the proposed approach reduces clustering errors in both controlled and field conditions by aligning neighborhood connectivity with the underlying angular sampling structure.

Conventional approaches that rely on a fixed Euclidean threshold assume a uniform spatial density, which is fundamentally inconsistent with the angular sampling nature of rotating LiDAR sensors, especially those with nonuniform beam distribution. The proposed method addresses this limitation by embedding sensor geometry directly into the clustering criterion, enabling consistent grouping decisions across near- and far-field regions.

The calibration results further indicate that clustering performance is governed by a structured trade-off between preserving complete vehicle clusters and preventing the merging of neighboring vehicles. The scaling factor λ was observed to define a stable operating region, with minimal error achieved within a bounded interval. This indicates that the proposed formulation is not overly sensitive to parameter variations, which is a desirable property for deployment in real-world systems. Similarly, the role of Δr was found to be secondary but important for stability. Although higher values yielded slightly lower error under controlled conditions, a more conservative choice was necessary to prevent cluster merging in dense traffic environments.

An important observation from the results is that the dominant error mode in conventional clustering is not over-segmentation but rather the failure to form valid clusters in sparse regions. As demonstrated in Section 4, fixed-threshold methods frequently produce missed detections in the far-field due to insufficient connectivity, leading to an increase in false negatives. The proposed formulation mitigates this issue by scaling the distance threshold with the radial distance and beam separation. Accordingly, it allows distant objects to remain structurally coherent without artificially increasing connectivity in dense regions.

The evaluation of intersection datasets further indicates that the proposed approach can maintain or improve clustering performance under more complex field conditions. Despite challenges such as occlusion, variable traffic density, and environmental noise, the geometry-based threshold outperformed the baseline approach on the evaluated datasets and maintained stable clustering behavior without parameter retuning within the same sensor configuration.

Although the results are promising, the method remains dependent on sensor-specific characteristics. The threshold formulation is general, but its numerical implementation depends on the vertical beam distribution, angular resolution, mounting height, ROI limits, and calibrated values of λ and Δr. For different LiDAR models or deployment settings, the threshold matrix should be recomputed from the corresponding geometry.

In addition, previous roadside LiDAR studies have shown that azimuth or horizontal angular information is also part of the sensor-coordinate representation used to model perception and point-cloud organization [5,9]. Therefore, future work should further evaluate how azimuth-dependent effects influence the proposed threshold formulation under different roadway layouts and sensor placements. Cross-sensor and cross-configuration validation will be considered in future work.

Finally, the computational overhead introduced by the proposed framework is negligible, as evidenced by the results summarized in Table 2. Incorporation of the threshold matrix *T* relies on a precomputed lookup structure, which replaces expensive geometric computations with simple distance normalization and indexing operations. Consequently, the additional processing time remains minimal, with only a marginal increase in per-frame latency. Importantly, the use of hierarchical clustering further supports computational efficiency in this context, as it naturally integrates with the normalized distance metric without requiring iterative density estimation or complex neighborhood searches. This combination demonstrates that hierarchical clustering is a suitable and efficient backbone for the proposed approach, enabling improved clustering performance while maintaining real-time feasibility.

## 6. Conclusions

This study presented a geometry-aware clustering formulation for roadside LiDAR data that directly incorporates the angular sampling structure of the sensor into the neighborhood criterion. Clustering tolerance is derived from both the angular sampling and the deployment geometry, modeling neighborhood connectivity as a function of vertical beam separation and sensing distance. This results in an adaptive threshold that remains physically consistent across the entire sensing range.

The resulting threshold matrix was integrated into existing clustering frameworks, including DBSCAN and hierarchical clustering, through normalized distance evaluation. This strategy enables adaptive thresholding without requiring structural modifications of the underlying algorithms. Experimental results demonstrated that the method consistently improves clustering performance in both controlled and operational scenarios. Specifically, the proposed formulation reduces false negatives in sparse regions while avoiding excessive merging in dense regions through controlled threshold scaling. The calibration analysis showed that the method operates within a stable parameter range, indicating robustness to moderate variations in λ and Δr.

The evaluation on field datasets further confirmed the scalability and generalization capability of the approach, achieving higher accuracy than the baseline method without the need for parameter retuning. In addition, the integration of the threshold matrix introduces negligible computational overhead, enabling real-time deployment. The results also highlight that hierarchical clustering provides an effective and efficient backbone for the proposed framework.

These findings suggest that infrastructure-based LiDAR systems benefit significantly from association rules that reflect the physics of the sensing process. Specifically, the ability to resolve sparse clusters at greater distances makes this method highly suitable for high-speed corridor applications, enabling the early detection required for proactive safety warnings. Future work will explore the integration of the adaptive criterion with learning-based perception frameworks to improve feature representation in sparse regions. Additional efforts will focus on optimizing computational efficiency for large-scale urban applications and developing adaptive parameter selection strategies to extend the method to diverse LiDAR configurations and highly dynamic multi-object environments.

## Figures and Tables

**Table 1:** Overview of notations for variables and parameters.

Notation	Description
$\alpha_{i}$	Horizontal angle of the *i*-th azimuth sample, expressed in radians
$\omega_{j}$	Vertical angle of the *j*-th LiDAR beam, expressed in radians
$N_{\alpha }$	Number of horizontal angular samples per scan
$N_{\omega }$	Number of vertical LiDAR beams
$\Delta \alpha_{i}$	Difference between consecutive horizontal angles
$\Delta \omega_{j}$	Difference between consecutive vertical beam angles
*r*	Radial distance from the sensor
$r_{m}$	Representative value of the *m*-th radial bin
$d_{m}$	Upper or representative bound of the *m*-th distance bin
Δr	Local radial difference between neighboring points
$d_{\mathrm{geom}}$	Local geometric spacing induced by LiDAR sampling
$h_{L}$	LiDAR mounting height
$z_{\mathrm{ROI}}^{\max}$	Upper vertical boundary of the region of interest
$z_{\mathrm{ROI}}^{\min}$	Lower vertical boundary of the region of interest
$r_{\mathrm{ROI}}$	Maximum sensing radius of the region of interest
$r_{j}^{\max}$	Maximum valid radius associated with beam *j*
$r_{j,m}^{\mathrm{eff}}$	Effective radius for beam *j* at radial bin *m*
λ	Scaling factor
$P_{\min}$	Minimum number of points required for a cluster
$t_{j,m}$	Threshold associated with beam *j* and radial bin *m*
$T_{j,m}$	Aggregated threshold for beam *j* and radial bin *m*
$T_{g,m}$	Threshold matrix entry for angular group *g* and radial bin *m*
$G_{\omega }$	Set of angular groups obtained from vertical-angle discretization
$d(p_{i},p_{k})$	Euclidean distance between points $p_{i}$ and $p_{k}$
$d_{\mathrm{norm}}$	Normalized pairwise distance

**Table 2:** Performance comparison of geometry-dependent clustering configurations.

Method	Scaling Factor	Δr	Accuracy (%)	Comp.Time per Frame (ms)	Detected Clusters
Hierarchical Clustering (Baseline)			95.64%	4.67	9893
Hierarchical Clustering	1.9	0.4	99.44%	4.98	9815
Hierarchical Clustering	1.7	0.4	99.60%	4.84	9833
DBSCAN (Baseline)			85.22%	4.94	11,387
DBSCAN	1.9	0.4	97.38%	6.64	9762
DBSCAN	1.7	0.4	97.38%	7.12	9762

**Table 3:** Tracking performance comparison between classical and geometry-aware clustering methods.

Method	λ	Δr	MOTA	FN	FP	IDSW	GT
Hierarchical Clustering (Baseline)			0.9849	113	29	6	9800
Hierarchical Clustering	1.7	0.4	0.9969	27	3	0	9800
DBSCAN (Baseline)			0.7664	1060	1239	19	9800
DBSCAN	1.7	0.4	0.9598	304	70	20	9800

## Data Availability

Data will be made available upon request.

## Supplementary Materials

The following supporting information can be downloaded at: https://www.mdpi.com/article/10.3390/s26134068/s1, Supplementary File S1: Dataset and code used to produce Figure 3 and Figure 10. The ZIP file includes the generated figure, a README file, tracking result files in JSON format for the classic hierarchical clustering method and the proposed GA-based approach, and a Python script (Python 3.8) for reproducing the tracking performance visualization.

## Abbreviations

The following abbreviations are used in this manuscript:

LiDAR	Light detection and ranging
DBSCAN	Density-based spatial clustering of applications with noise
FSPC	Fast-spherical-projection-based clustering
ROI	Region of interest
MARE	Mean Absolute Relative Error
MOTA	Multiple Object Tracking Accuracy
