# Supplementary figures and images for: Spatial Geometry Analysis of Roadside LiDAR for Improved Vehicle Clustering Accuracy
Source: Sensors (Basel). 2026 Jun 26;26(13):4068. doi: 10.3390/s26134068 (PMC13363753; doi:10.3390/s26134068)

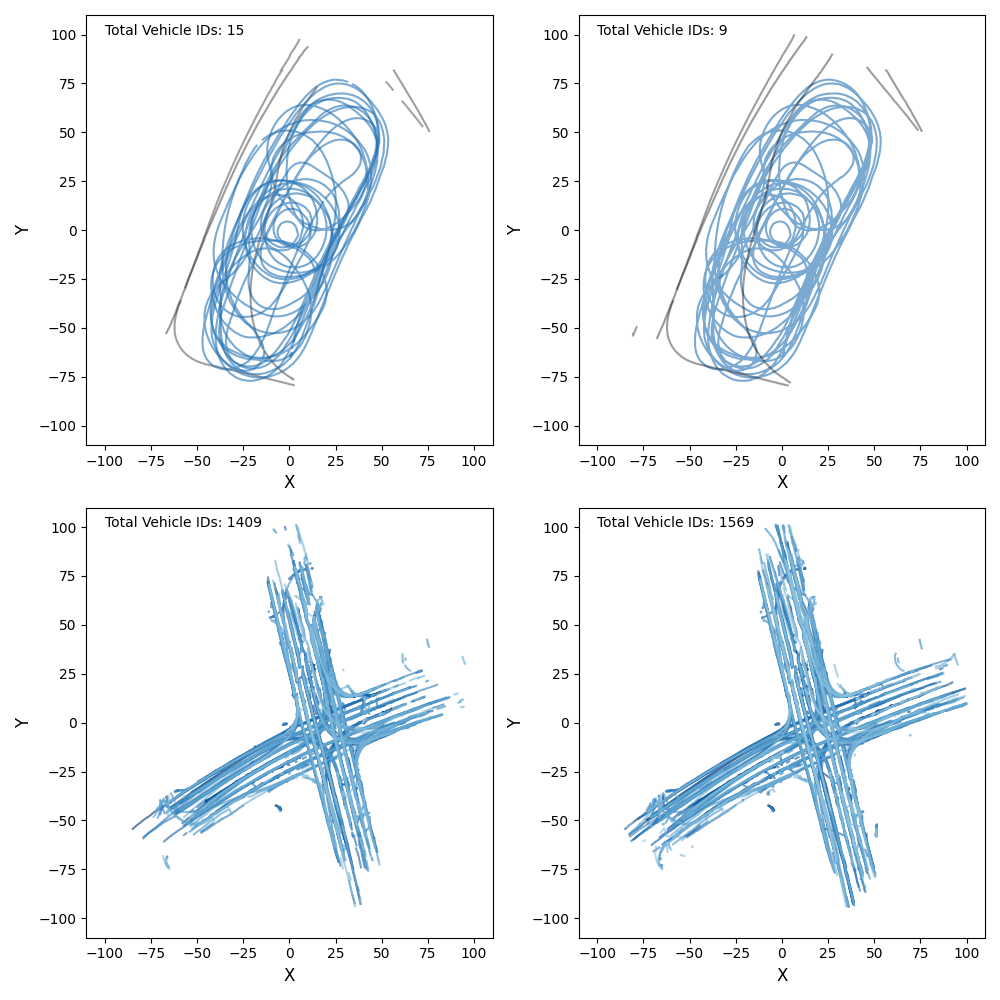

Supplement: Supplementary file 1 [file sensors-26-04068-s001.zip › Figure_3_and_10.png]
